# Genetics of ADHD: What Should the Clinician Know?

**DOI:** 10.1007/s11920-020-1141-x

**Published:** 2020-02-27

**Authors:** Oliver Grimm, Thorsten M. Kranz, Andreas Reif

**Affiliations:** Department of Psychiatry, Psychosomatic Medicine and Psychotherapy, University Hospital, Goethe University, Frankfurt, Germany

**Keywords:** ADHD, Genetics, Polygenic risk score, Attention deficit, Hyperactivity, Genetic syndromes, Copy number

## Abstract

**Purpose of Review:**

Attention deficit hyperactivity disorder (ADHD) shows high heritability in formal genetic studies. In our review article, we provide an overview on common and rare genetic risk variants for ADHD and their link to clinical practice.

**Recent findings:**

The formal heritability of ADHD is about 80% and therefore higher than most other psychiatric diseases. However, recent studies estimate the proportion of heritability based on singlenucleotide variants (SNPs) at 22%. It is a matter of debate which genetic mechanisms explain this huge difference. While frequent variants in first mega-analyses of genome-wideassociation study data containing several thousand patients give the first genome-wide results, explaining only little variance, the methodologically more difficult analyses of rare variants are still in their infancy. Some rare genetic syndromes show higher prevalence for ADHD indicating a potential role for a small number of patients. In contrast, polygenic risk scores (PRS) could potentially be applied to every patient. We give an overview how PRS explain different behavioral phenotypes in ADHD and how they could be used for diagnosis and therapy prediction.

**Summary:**

Knowledge about a patient’s genetic makeup is not yet mandatory for ADHD therapy or diagnosis. PRS however have been introduced successfully in other areas of clinical medicine, and their application in psychiatry will begin within the next years. In order to ensure competent advice for patients, knowledge of the current state of research is useful forpsychiatrists.

## Introduction

Attention deficit hyperactivity disorder (ADHD) is a developmental disorder with symptoms of inattentiveness, impulsiveness, and hyperactivity, which leads to impairments in everyday life and manifests before the age of 12. The developmental trajectory shows a typical course of clinical symptoms, e.g., decrease of hyperactivity, occurrence of comorbid disorders, e.g., addiction and depression, as well as economic costs and social impairments [1]. ADHD has a worldwide prevalence of 5 to 7% of school-age children [2]. In childhood, impulsiveness and hyperactivity are leading symptoms, but decrease in adulthood, whereas inattentiveness becomes the leading symptom [3]. The extent to which symptoms completely remit or persist into adulthood is variable. While the cross-sectional prevalence of ADHD in adults was estimated between 2.5 and 3% [4], the persistence of symptoms with corresponding impairments into adulthood is about 65%.

Early on, clinicians noticed that ADHD-typical behavior occurs frequently in syndromic disorders, e.g., Klinefelter syndrome, Williams syndrome, fragile X syndrome, or tuberous sclerosis [5]. The high heritability [6] suggests a significant genetic component (see below). Genetic research can contribute not only to the elucidation of neurobiological mechanisms but also to clinical questions such as the effect of operationalization or the genetic correlation with comorbid disorders. Patients with ADHD show high comorbidity with autism, obesity, bipolar disorder and depression, anxiety, and substance use disorder [1, 7]. This suggests common underlying risk gene variants. Genetic correlations provide insights how biologic mechanisms manifest in different but related disorders (pleiotropy). In the following, we focus on general aspects of heredity and the complementary results of the analysis of common and rare variants of the genome.

## Heritability in ADHD

There are several ways to investigate the heritability of ADHD. A classical strategy makes use of twin studies, due to the possibility of assessing the genetic effect (heritability) of the disorder. According to a recent meta-analysis of twin studies, the heritability of ADHD is estimated at 77–88% [8]. The magnitude is therefore similar to that of autism spectrum disorder (about 80%), bipolar disorder (about 75%), and schizophrenia (about 80%) [6].

By means of genome-wide complex trait analysis (GCTA), thousands of individuals are examined for hundreds of thousands of single-nucleotide polymorphisms (SNPs) and thus provide a measure of heritability, the so-called SNP-based heritability. A recent mega-analysis estimates SNP-based heritability (h^2^ snp = 22%) in a range comparable to previous estimates of h^2^ snp for ADHD in studies with fewer subjects (h^2^ snp, 10–28%) [9]. The proportion of heritability that explains this gap between approximately 74% in twin studies and 22% in SNP-based heritability is also referred to as “hidden heritability” in reference to the search for dark matter in astronomy. One explanation is the fact that statistical power of the performed genome-wide association studies (GWAS) is too small to reliably predict genetic associations. A core problem is the required number of test persons in order to reliably map a genetic association, preferably one with a high effect size. A higher number of participants, along with other measures such as the refinement of statistical methods, would certainly help to considerably increase the predictive power of GWAS. A stronger effect size of the SNPs, a higher allele frequency of the rare alleles of SNPs, and a higher LD lower the required number of subjects decisively [10, 11] which in turn means that, in ADHD, there are either very rare alleles or low effect sizes in action given as the latest and largest GWAS only gave 12 genome-wide significant hits by examining more than 20,000 cases.

In addition to the relationship between the number of subjects and the allele frequency of the SNPs, other DNA variants, such as copy number variations (CNV), may explain the missing heritability. CNVs are sections of DNA that either occur in multiple copies or deletions of certain chromosomal sections. CNVs occur at different frequencies but are quite common in patients with ADHD. Small CNVs of only a few kilobases are not detected with the necessary accuracy by GWAS, yet make up the considerably larger part of the genetic variability in ADHD. Large CNVs (> 500 kb) with a frequency of less than 1% can only be detected using sufficiently large sample sizes. In order to be able to map all possible CNVs, whole-genome sequencing (WGS) would be the method of choice.

## Genome-Wide Association Studies (GWAS)

Until the late 2000s, candidate gene studies on small samples were the method of choice for testing genetic associations. Genes coding for components of the monoamine transmitter systems was first investigated in ADHD. However, none of the candidate genes was replicated by larger genome-wide studies. A summary of these studies can be found in authoritative reviews [8, 12, 13]. While GWAS initially identified only a few loci for psychiatric disorders [14], the most recent collaborations succeeded in discovering a larger number of genome-wide significant loci (*p* ≤ 5 × 10^−8^) by significantly increasing the number of cases and control numbers (> 10,000).

A study of 909 parent-child trios with ADHD-affected children revealed strong genetic associations of the genes glucose-fructose oxidoreductase domain-containing 1 (*GFOD1*) and cadherin 13 (*CHD13*) [15–17] with ADHD. *GFOD1* is expressed in the frontal cortex; the exact function of the gene is not yet known [18]. *CDH13* encodes for a calcium-dependent cell-cell adhesion protein that influences neuronal development and synaptic plasticity [22]. In a knockout mouse model of *Cdh13*, mice showed hyperlocomotion and learning deficits [19]. These findings, together with evidence from other association studies in which *CHD13* is associated with, e.g., autism, schizophrenia, bipolar disorder, and depression [20], make *CDH13* an interesting candidate gene for ADHD.

Another ADHD candidate gene found in a large family by linkage analysis and replicated in parallel in a global case-control study (*n* = 2627 ADHD subjects, *n* = 2531 controls) is adhesion-G protein-coupled-receptor-L3 (*ADGRL3*, formerly *LPHN3*), a brain-specific G protein-coupled receptor with cell adhesion function [23]. *ADGRL3* was confirmed as an ADHD candidate locus in two other independent case-control studies, by association of one haplotype in *ADGRL3* [21] and single associations of several SNPs [22]. In the zebrafish model, the loss of *adgrl3* leads to a reduction of dopaminergic neurons in the ventral diencephalon and a hyperactive/impulsive phenotype [23], whereas in *Adgrl3*-knockout mice, an increase in reward motivation and activity level as well as other ADHD-analogous behaviors was observed—parallel to dysregulation of the dopamine transporter [24, 25]. This suggests that the biological validation of an ADHD candidate gene from a GWAS in an animal model can elucidate potential mechanisms of pathogenesis. On the other side, it must be critically questioned whether behavioral traits such as hyperlocomotion in animals are equivalent ADHD-hyperactivity in humans.

In the most recent and comprehensive meta-analysis of ADHD GWAS data sets to date (20,183 cases, 35,191 controls), 12 genomic loci with genome-wide significance (*p* < 5 × 10^−8^) could be identified [26•]. The 12 loci cover 16 genes, of which at least four have a high expression in the human brain; for example, *DUSP6* regulates the synaptic transmitter dopamine, while *MEF2C* is primarily associated with autism and intelligence impairment [8]. Furthermore, there was a genetic correlation with achieved school and education goals, i.e., people with a higher genetic risk for ADHD have a slightly lower success in school and education, regardless of the diagnosis ADHD. Similarly, an earlier study argued for a high genetic correlation (*r* = 0.64) of ADHD and bipolar disorder [27].

A recent meta-analysis in more than 17,000 cases showed a high overlap in genetic correlation between children and adults as well as continuous measures as well as categorical ADHD definition [28]. There, genetic factors influencing ADHD persistence in adults (6532 patients with ADHD and 15,874 healthy controls) and early childhood ADHD (10,617 children with ADHD and 16,537 healthy controls) were compared. A comparison of the data sets showed a high genetic correlation (*r* = 0.81) between early childhood and adult ADHD arguing that these are indeed covering the same clinical phenotype. An additional meta-analysis of ADHD over the entire life span in children and adults revealed nine genes significantly associated with ADHD over the life span. This underscores how GWAS can contribute to clinical questions about differences between childhood and adult manifestations of ADHD.

## Rare Variants and genetic Syndromes

The association of genetic syndromes with ADHD in childhood has been known for a while. Chromosomal aberrations have been frequently found in ADHD and other developmental disorders [29].

Fragile X syndrome (FXS) is one of the most frequent genetic syndromes associated with ADHD. Based on parent or teacher reports, 59% of boys with FXS meet diagnostic behavioral criteria for either ADHD-inattentive type only (31.5%), ADHD-hyperactive type only (7.4%), or ADHD-combined type (14.8%) [5]. FXS is caused by loss-of-function of the *FMR1* gene, which encodes the RNA-binding protein, called fragile X mental retardation protein (FMRP). Exaggerated glutamatergic excitation and reduced GABA-signaling have been discussed as main mechanisms for FXS.

Neurofibromin 1 (*NF1*) is characterized by different tumors in the eyes, the skin, and the central nervous system. The *NF1* locus is situated on chromosome 17q11.2. About one third is accompanied by ADHD symptoms during childhood [30]. The neurobiological basis of the link between ADHD and *NF1* might stem from lesions in the basal ganglia.

Tuberous sclerosis complex (TSC) is an autosomal dominant hereditary disorder associated with brain malformations and tumors, skin lesions, and mostly benign tumors in other organ systems, often clinically characterized by epileptic seizures and cognitive impairment. ADHD prevalence in TSC patients has been ranging from about 30 to 60% [31•].

The sexual aneuploidies Turner syndrome and Klinefelter syndrome have been associated with ADHD as well. In patients suffering from Klinefelter syndrome, the rate of ADHD is as high as 63% [32]. In our experience, these patients are more often seen by adult psychiatrists than the aforementioned syndromes.

Williams-Beuren syndrome (WBS) is a microdeletion on chromosome 7 and is associated with a typical set of symptoms ranging from special facial features (“elf-like”) to pulmonal and cardiovascular anomalies. On a behavioral level, they are characterized as being “hypersocial.” Almost two thirds of WBS show symptoms of ADHD [33]. The deletion of *Limk1* leads to hyperactivity and impaired spatial learning in knockout mice.

Velo-cardio-facial/DiGeorge syndrome (22q11 deletion syndrome) is associated with cardiovascular abnormalities and a variety of psychiatric disorders [34]. Previous studies often focused on schizophrenia. However, much more patients suffer from ADHD (about 40%), most of them from the inattentive type [35]. The hemizygous 22q11 deletion encompasses the *COMT* gene which is a bottleneck for the catecholamine neurotransmission.

While these syndromes are rare, they should be known to the adult psychiatrist because most patients come into contact with the medical system due to the complex comorbidities, e.g., heart disorder in 22q11. However, there are no studies looking at the behavioral trajectory of these genetic syndromes and associated ADHD in adults. Nevertheless, recent polygenic risk analyses suggest that a significant part of the heredity of ADHD is caused by rare variants [36]. Rare variants do not show the high penetrance of deleterious variants of syndromic disorders. While it is likely that their effect size or penetrance is much lower, their frequency might be more common than the syndromic disorders mentioned above. Some of the “missing heritability” might be explained by these rare variants as the effects of rare variants can account for up to one quarter of heritability [14, 26•]. The largest classes of rare variants are single-nucleotide variants (SNVs), i.e., classical although very rare polymorphisms (minor allele frequency < 0.1%) of one base pair, and copy number variants (CNVs). CNVs are duplications or deletions and contain both coding and non-coding DNA. While a single variant is rare by definition (0.3–1% of the total human genome), CNVs represent up to 10% of the human genome. The effects of such copy number polymorphisms (CNPs) on ADHD risk have not yet been investigated in detail, although there is a first study [37] that shows an association of a specific CNP with ADHD.

A study in more than 2800 children with ADHD found an accumulation of large and rare CNVs in the 15q13.3 locus. Although still rare, the frequency of 0.6% results in a relatively high effect size with an odds ratio of 2.2, which is significantly higher than any single locus in ADHD-GWAS [29].

Initial studies examining CNVs in ADHD case samples focused on children but not on adults. However, due to the small sample size and evolving methodology, the results are inconsistent. A British and an Icelandic study of children found a disproportionately high level of rare, large CNVs in ADHD patients compared to healthy controls [38, 39]. Duplications on chromosome 16p13.11 were observed, as well as loci which occurred in autism and schizophrenia.

Further genome-wide studies on ADHD in childhood investigated the overall burden of rare, large CNVs in patients compared to healthy subjects. They provide some evidence of an accumulation of rare variants in childhood ADHD [40–42]. Nevertheless, there is no ADHD-specific CNV, but an increase in the total number of CNVs within ADHD patients (“high CNV burden”) compared to the control group is observed.

In a genome-wide screening for CNVs in a cohort of 99 children and adolescents with severe ADHD, seven syndrome-associated CNVs were identified. The gene coding for neuropeptide Y (*NPY*) had been deleted in a ∼ 3 Mb duplication on chromosome 7p15.2–15.3. Interestingly, this was associated with an increased NPY plasma concentration in affected family members [43].

In a larger whole-genome CNV study with 1013 cases of ADHD and 4105 healthy children of European descent, CNVs affecting metabotropic glutamate receptor genes were enriched across all cohorts [44]. The study identified *GRM5* (coding for glutamate receptor, metabotropic 5) deletions in ten cases and one control, *GRM7* deletions in six cases, and *GRM8* deletions in eight cases and no controls.

An innovative approach was used in a linkage analysis of three German ADHD families (*n* = 70), where rare variants in the regions inherited by affected members were tested in a large (*n* > 9000) independent cases and controls sample. This led to the identification of *AAED1,* which interacts with the protein kinase C-alpha-binding protein (*PICK1*). *PICK1* is a regulator of dopamine transporter trafficking dopaminergic neurons.

Since CNVs are rare, these initial results must be considered with caution. The role of these rare variants in healthy populations is not yet clear, and their frequency and location are currently being finalized and mapped. Depending on the definition, a recent CNV map estimated that 4.8–9.5% of the genome contributes to CNVs. This mapping identified about 100 genes that can be completely deleted without producing an apparent phenotype [45]. Therefore, caution in interpreting associations between ADHD and CNVs of unknown function is needed.

Another group of genetic variations, the so-called single-nucleotide variants (SNVs), also seems to have a large share in the unexplained genetic variability of ADHD. A recent study with 123 ADHD patients and 82 healthy controls by means of complete exome sequencing shows that within the group of ADHD patients, a strongly increased number of amino acid altering SNVs with rare allelic frequency (< 0.1%) are observed [46]. Another study compared the results of an ADHD case control study on de novo and rare SNVs with those of autism cohorts. It was found that autism and ADHD patients carry a similarly high SNV load (“SNV burden”), which leads to shortened and potentially functionally altered proteins [47].

Should the knowledge about these rare variants lead to clinical sequencing tests in ADHD patients? So far, this cannot be recommended as these variants still seem to be rare, and complete genome sequencing is expensive. However it can be considered in patients with comorbid intellectual disability.

## Polygenic Risk Scores in ADHD and ADHD-Related Traits

A polygenic risk score (PRS) uses the summary statistics of SNP results from large GWAS to predict clinically significant variables. The idea of the PRS is to provide genetic risk prediction, given the large set of SNPs for each individual, and use it as a predictive tool for a specific trait [48•]. An individual PRS can be calculated by summing all trait-associated SNPs weighted by their effect sizes [49]. In addition, PRS can be calculated for single phenotypes that are related to ADHD. As this technique has gained some popularity, we will give an overview of its application in ADHD and related psychiatric disorders.

PRS provide interesting insights into the dimensional structure of ADHD: A PRS of ADHD risk variants is correlated with attention deficits in the general population [50] supporting the notion that ADHD is not a clear-cut categorical disorder but the extreme end of a genetically determined, continuous behavioral trait. In addition to an approach generalizable to non-diseased participants, the impact of PRS on ADHD-related endophenotypes [51] is supported by neuroimaging studies. Genetic variants related to intelligence and education are positively associated with larger total brain volumes in children. However, genetic variants associated with ADHD were related to smaller caudate volume, a subcortical region of the brain that has been consistently found to be reduced in individuals affected by this disorder [52].

Apart from these more theoretical insights, PRS can be applied to more clinical questions: ADHD comes with a high burden of comorbid disorders which can be studied in large epidemiological samples like the UK Biobank sample. In more than 135,000 participants, an ADHD PRS was highly linked to depression, anxiety, alcohol intake, risk-taking, and negatively to verbal-numerical reasoning. While this gives general insight into the genetic structure of these traits, it should be kept in mind that ADHD and other disorders are probably underdiagnosed in this large sample [53•].

ADHD in childhood, but in some parts in adulthood, too, is tightly linked to school achievement. In studies looking at the link between ADHD PRS and educational achievement or even intelligence, it is clear that PRS predicts educational achievement [26•]. Interestingly, this link is not restricted to ADHD cases but generalizes to the normal population [54].

PRS can even contribute to the highly debated question, what defines a “persister” versus a remitter (from childhood ADHD) in patients? A recent population-based cohort correlated an ADHD PRS to ADHD symptom trajectories between ages 4 and 17 [55]. The ADHD PRS was higher in the persistence group in children, pointing to the effect of genetics in the course of the disorder.

Studies using a PRS from other psychiatric disorders, e.g., a schizophrenia risk PRS, predicted ADHD and oppositional defiant disorder in a large sample of children (ALSPAC) [56]. PRS scores from multiple psychiatric disorders can be used to enhance the discriminatory power between them. Using PRS for five psychiatric disorders and imaging data on neural connectivity, it was shown that there are shared altered functional connectivity patterns for autism spectrum disorder, bipolar disorder, and schizophrenia versus ADHD [57].

Another study investigated the relationships of PRS of psychiatric disorders and substance (ab-)use. Using PRS for cross-disorder psychopathology (CROSS) from 2573 European-American participants and information on liability to alcohol, cannabis, cocaine, nicotine, and opioids, the authors identified negative association with cannabis and positive association with nicotine use for ADHD that did not survive after correction for multiple testing; however, in the other psychiatric disorders, statistically relevant patterns between substance and disorder-specific PRS could be identified [58]. These studies underscore that related but distinct genetic risk contributes to common patterns of developmental psychopathology.

In a study which looked at socioeconomic variables like employment, individual income, and household wealth, an ADHD PRS was associated with more negative outcomes in these variables and increased the likelihood of receiving social security disability benefits, unemployment or worker compensation [59].

However, application of PRS in clinical practice comes with some drawbacks, e.g., most GWAS concentrate on populations of European ancestry. The application in other areas of clinical medicine led to severe problems in interpretation of results [60•].

While these applications of PRS for understanding ADHD are exciting, it should be kept in mind that PRS typically explain only about 5.5% in variance [26•]. In an exemplary study of height, SNPs in a sample of about one million genotyped participants explained 48% variance of body height [61]. Nevertheless, the receiver-operator accuracy for correctly predicting height from a PRS was between 55 and 65%. This is much too low for a simple clinical screening test and even more applies to ADHD, as the primary studies are far smaller and underpowered.

## Discussion

The findings of recent years indicate that there is a specific genetic basis for ADHD in children and adults that not only increases the risk of the disorder but also the risk of other independent psychiatric diagnoses and socially relevant measures such as school and learning outcomes [26•]. How can this new knowledge be applied to support treatment and diagnosis? In terms of diagnosis, a first step could be to identify rare variants in adults and children that could help distinguish them from other genetic syndromes or neurological disorders. However, this will require even greater validation of the findings. For most patients, the common variants do not seem to explain enough variance to be used in predicting diagnosis. This may change if gene set analyses or polygenic risk scores allow a substantial explanation of heredity.

A general finding from the last 20 years of ADHD psychiatric genetics is that risk genes involved in the regulation of catecholaminergic genes have not been reliably replicated. Genetic findings show that ADHD is more than a simple “catecholaminergic disorder.” The genome-wide loci affected by ADHD often have a general function in areas such as “neurite outgrowth,” “synaptic plasticity,” or “glutamatergic signal transmission” [26•, 62, 63]. Research over the next few years must show whether the common denominator of these variants is brain expression and regulation of neuronal activity and development or whether there are completely different underlying causes [64].

Another field that could see an earlier clinical application of genetics is the prediction of pharmacotherapy, the so-called pharmacogenomics. Initial studies on pharmacogenomics in ADHD looked at common candidate genes such as the dopamine transporter [65]. Since therapy is mainly based on improvement of dopaminergic neurotransmission, it is plausible to expect a stronger effect of genes involved in the mediation of dopaminergic effects. Smaller studies have shown that polymorphisms in genes of catecholaminergic neurotransmission [66] or the SNARE complex of the synapse can indeed predict the response to stimulant therapy [67].

## Conclusion

The application of genomic methods for differential or primary diagnosis of psychiatric disorders does not play a role in clinical medicine so far. Since the explained variance is still too low, other factors for therapy prediction and diagnosis will play a greater role in individual cases. At present, patient treatment does not focus on genetic testing but on an exact clinical diagnosis. Nevertheless, in the future, psychiatrists will have to deal with the fact that some of their patients are diagnosed with a rare variant (SNV or CNV). Currently, it can be stated that beyond already defined genetic syndromes (e.g., 22q11-DS), there is no proven rare variant that determines or predicts ADHD.

PRS are not yet helpful in differential diagnosis either; however, it is to be expected that this will change in the upcoming years. For example, the PRS for breast cancer has been combined with conventional risk factors to identify 16% of the population who may benefit from an earlier screening (and 32% who may delay screening) [68]. In coronary heart disease, PRS have identified individuals with a risk who benefit more from early initiation of statin therapy than individuals with a lower genetic risk [69]. It can be assumed that similar results will also find their way into psychiatry, even though genetic investigations in ADHD are currently of predominantly scientific and not yet clinical significance.

In general, the genetic disposition plays a major role in the pathogenesis of ADHD. On the one hand, knowledge and public discussion about this can counteract stigmatization of patients. Genetic causes are now accepted by many affected patients and their relatives as an explanatory model [70, 71]. An increase in knowledge of genetics and neurobiology will not replace the physician’s intuition in diagnosing and treating the individual patient. Psychiatric genetics will not change the art of clinical medicine, i.e., the way physician and patient communicate about mental health, but it will provide a useful tool for a more personalized medicine.
